# Vascular Deformation Mapping for CT Surveillance of Thoracic Aortic Aneurysm Growth

**DOI:** 10.1148/radiol.2021210658

**Published:** 2021-10-19

**Authors:** Nicholas S. Burris, Zhangxing Bian, Jeffrey Dominic, Jianyang Zhong, Ignas B. Houben, Theodorus M. J. van Bakel, Himanshu J. Patel, Brian D. Ross, Gary E. Christensen, Charles R. Hatt

**Affiliations:** From the Departments of Radiology (N.S.B., Z.B., J.D., J.Z., B.D.R., C.R.H.), Biomedical Engineering (N.S.B.), and Electrical Engineering and Computer Science (Z.B., J.D., J.Z.), Center for Molecular Imaging (N.S.B., B.D.R.), and Departments of Cardiac Surgery (I.B.H., T.M.J.v.B., H.J.P.), and Biological Chemistry (B.D.R.), University of Michigan, 1500 E Medical Center Dr, CVC 5588, SPC-5030, Ann Arbor, MI 48109-5030; Department of Electrical and Computer Engineering, University of Iowa, Iowa City, Iowa (G.E.C.); and Imbio, Minneapolis, Minn (C.R.H.).

## Abstract

**Background:**

Aortic diameter measurements in patients with a thoracic aortic aneurysm (TAA) show wide variation. There is no technique to quantify aortic growth in a three-dimensional (3D) manner.

**Purpose:**

To validate a CT-based technique for quantification of 3D growth based on deformable registration in patients with TAA.

**Materials and Methods:**

Patients with ascending and descending TAA with two or more CT angiography studies between 2006 and 2020 were retrospectively identified. The 3D aortic growth was quantified using vascular deformation mapping (VDM), a technique that uses deformable registration to warp a mesh constructed from baseline aortic anatomy. Growth assessments between VDM and clinical CT diameter measurements were compared. Aortic growth was quantified as the ratio of change in surface area at each mesh element (area ratio). Manual segmentations were performed by independent raters to assess interrater reproducibility. Registration error was assessed using manually placed landmarks. Agreement between VDM and clinical diameter measurements was assessed using Pearson correlation and Cohen κ coefficients.

**Results:**

A total of 38 patients (68 surveillance intervals) were evaluated (mean age, 69 years ± 9 [standard deviation]; 21 women), with TAA involving the ascending aorta (*n* = 26), descending aorta (*n* = 10), or both (*n* = 2). VDM was technically successful in 35 of 38 (92%) patients and 58 of 68 intervals (85%). Median registration error was 0.77 mm (interquartile range, 0.54–1.10 mm). Interrater agreement was high for aortic segmentation (Dice similarity coefficient = 0.97 ± 0.02) and VDM-derived area ratio (bias = 0.0, limits of agreement: −0.03 to 0.03). There was strong agreement (*r* = 0.85, *P* < .001) between peak area ratio values and diameter change. VDM detected growth in 14 of 58 (24%) intervals. VDM revealed growth outside the maximally dilated segment in six of 14 (36%) growth intervals, none of which were detected with diameter measurements.

**Conclusion:**

Vascular deformation mapping provided reliable and comprehensive quantitative assessment of three-dimensional aortic growth and growth patterns in patients with thoracic aortic aneurysms undergoing CT surveillance.

Published under a CC BY 4.0 license

*Online supplemental material is available for this article.*

See also the editorial by Wieben in this issue.

## Graphical abstract

**Figure:**
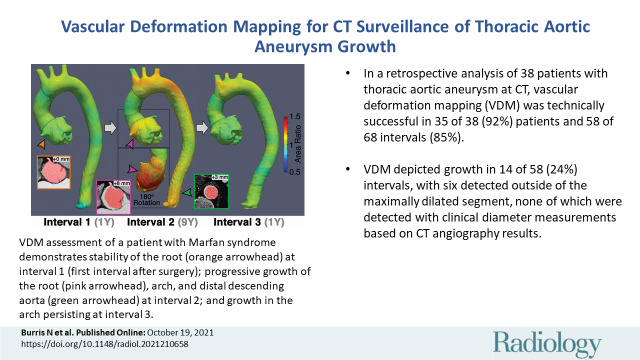

**Summary**

Vascular deformation mapping, a deformable image registration-based technique, enabled reliable comprehensive assessment of the degree and extent of three-dimensional growth among patients with a thoracic aortic aneurysm undergoing CT surveillance.

**Key Results**

■ In a retrospective analysis of 38 patients with thoracic aortic aneurysm on CT scans, vascular deformation mapping (VDM) was technically successful in 35 of 38 (92%) patients and 58 of 68 intervals (85%).
■ VDM was used to detect growth in 14 of 58 (24%) intervals, with six detected outside of the maximally dilated segment, none of which were detected with clinical diameter measurements based on results of CT angiography.
■ VDM-derived measurements of aortic surface area change had low interrater variability (bias = 0.0); peak area ratio values and diameter change showed strong agreement (*r* = 0.85, *P* < .001).

## Introduction

Thoracic aortic aneurysm (TAA) is common and is increasing in prevalence worldwide, with approximately 3% of patients older than 50 years having a dilated thoracic aorta (1–3) and recommended to undergo imaging surveillance (4). Most patients with TAA have an indolent disease course, with aortic growth occurring either slowly or not at all over a period of years or decades (5). However, life-threatening complications, such as aortic dissection and rupture, can occur in otherwise asymptomatic patients at presurgical aneurysm sizes (6,7), emphasizing the need for better techniques with which to assess disease progression, inform surgical candidacy, and predict complications. A fundamental limitation to improved management of TAA is the lack of image analysis techniques with which to accurately assess aortic growth. Current assessment techniques are based on measurements of maximal aortic diameter. However, the degree of variability associated with aortic diameter measurements (within 1–5 mm despite optimal measurement technique) frequently prevents confident assessment of disease progression at typical TAA growth rates (<1 mm per year) (8–11). Also, diameter measurements are inherently two dimensional and are performed in fixed anatomic locations; thus, they are unable to capture the three-dimensional (3D) nature of TAA growth.

To overcome these limitations, prior research has described the feasibility of a medical image analysis technique, termed vascular deformation mapping (VDM), in 3D assessment of aortic growth using deformable image registration techniques (12,13). This approach uses high spatial resolution and volumetric CT angiography data and allows for comprehensive quantification of aortic growth at all points on the aortic wall, avoiding the limitations of manual definition of measurement planes. Despite these advantages, registration errors can occur due to nonoptimized registration parameters (eg, regularization and similarity metrics) or factors that degrade image quality (eg, motion or streak artifacts), which in turn will result in VDM measurement errors. Thus, an evaluation of VDM in a clinical cohort of patients with TAA is needed to understand the reliability and clinical utility of this technique.

This study focused on two primary objectives: *(a)* to determine performance of the VDM algorithms in a cohort of patients with TAA undergoing imaging surveillance that included assessment of reproducibility and identification of sources of error in the analysis workflow and *(b)* to characterize unique patterns of 3D aortic growth observed in patients with TAA and to assess the agreement of VDM analysis with standard diameter measurements.

## Materials and Methods

### Patient Identification and Clinical Data Abstraction

All procedures were approved by the local institutional review board (HUM00133798) and were compliant with the Health Insurance Portability and Accountability Act. We used electronic medical records search software developed at our institution (EMERSE; University of Michigan) (14) to identify patients at our tertiary academic institution undergoing imaging surveillance of TAA in the pre- or postoperative setting with serial (two or more) CT angiograms covering the thoracic aorta between November 2006 and January 2020. Patients were excluded from analysis for non–electrocardiographically-gated acquisition, lack of thin-section (≤3 mm) reconstructions, poor aortic opacification (<200 HU at the ascending aorta), interval surgical aortic repair, or severe motion artifacts. Scans with mild motion-related blurring affecting only the aortic root were included if the proximal coronary arteries could be clearly visualized. A total of 50 patients meeting these criteria were identified at random. CT acquisition parameters are described in Appendix E1 (online). Clinical and demographic information was collected through chart review. Maximal diameter measurements of the thoracic aorta were recorded from clinical CT reports. Of note, aortic measurements at our center are performed in a 3D laboratory by trained technologists using standardized measurement protocols and a centerline measurement technique (4).

### Vascular Deformation Mapping

**Figure 1:**
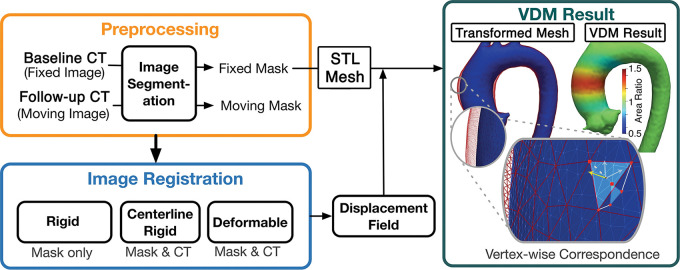
Simplified schematic overview of the steps involved in the vascular deformation mapping (VDM) analysis pipeline. Electrocardiographically gated aortic CT angiography Digital Imaging and Communications in Medicine data are retrieved for baseline and follow-up examinations, and CT angiography data undergo aortic segmentation (orange box), followed by rigid and deformable registration (blue box). The displacement field calculated from registration steps is used to translate the mesh vertices of the baseline model (blue surface) to the aortic geometry at follow-up (red mesh), and the ratio of change in the surface area of each mesh element (area ratio) is plotted on the aortic surface using a colorized scale. STL = stereolithography.

The VDM analysis pipeline for measurement of 3D aortic growth uses deformable image registration to quantify deformation of the aortic wall between two CT angiograms. The VDM analysis includes several steps: *(a)* segmentation of the thoracic aorta on CT angiography images from scans acquired at two different time points, with the first time point considered the fixed image and the second time point considered the moving image; *(b)* image preprocessing steps including cropping and clamping voxels with negative attenuation values (in Hounsfield units) at 0 to avoid the adjacent lung influencing the registration and dilation of aortic masks by three voxels to ensure inclusion of the wall; *(c)* rigid registration to approximately align the two CT angiographic images (Elastix 5.0.1; Utrecht University) (15); *(d)* implicit alignment of the aortic centerline using a highly regularized multiple-image multimetric deformable registration that applies a penalty term to enforce rigid movement of voxels within the aortic segmentation but allows deformation of the periaortic voxels (16); *(e)* multiresolution multimetric B-spline deformable image registration using mutual information with 10-mm grid spacing and a bending energy penalty of 100 (17); *(f)* generation of a polygonal mesh of the aortic surface at baseline (fixed) geometry; *(g)* translation of baseline aortic mesh vertices using the deformation field calculated in step 5; and *(h)* quantification of deformation as the ratio of surface area change at each triangular mesh element (termed area ratio) with color visualization in Paraview 5.9.0 (Kitware). VDM analysis takes approximately 20 minutes on a standard high-performance PC with parallelization. A simplified schematic overview of the VDM analysis pipeline is presented in Figure 1.

### Image Segmentation Technique and Interrater Reproducibility Analysis

Manual aortic segmentation was used in the VDM workflow to create aortic masks and is thus a potential source of variability. While all CT angiograms were segmented by a rater with 4 years of experience with aortic image analysis (I.B.H.), we had an additional rater with 5 years of experience (T.M.J.v.B.) perform segmentations on 45 randomly selected CT angiography intervals to investigate the influence of manual segmentation variability on VDM output. Raters segmented the thoracic aorta from the root to just beyond the celiac axis, including the proximal arch vessels, using segmentation software (Mimics, version 22.0; Materialise).

### Quality Assurance Process and Registration Accuracy Assessment

We adopted a multistep quality assurance protocol to evaluate the validity of each VDM output, with quality assurance steps performed by a researcher with 15 years of experience with cardiovascular CT (N.S.B.). The quality assurance protocol involved visual confirmation of segmentation and registration accuracy using dual-color plots to ensure overlap of the aortic wall after the final deformable registration step; specific steps in the quality assurance protocol are described in Appendix E1 (online).

To assess registration accuracy, landmarks were manually placed along the aortic wall by a senior researcher with 15 years of cardiovascular CT experience (N.S.B.). Landmark registration error was determined by calculating the Euclidean distance between homologous points after deformable transformation. Conserved anatomic landmarks, such as branch points and intimal calcifications, were used to place aortic landmarks across serial CT angiograms. Deformable registration was performed using VDM parameters in both the forward and the reverse direction and using all possible combinations of CT intervals for each patient.

### Statistical Analysis

Continuous variables are reported as mean ± standard deviation for normally distributed data, as median and interquartile range (IQR) for nonnormal continuous variables, and as frequencies for categorical variables. Normality was assessed using the Shapiro-Wilk test. Pearson correlation coefficient was used to assess correlation between continuous variables. Binary categories were created based on published data on reducibility of clinical diameter measurements (8–10), with growth defined as diameter change in the aneurysmal segment of at least 3 mm based on clinical measurements and at least 1.2 area ratio change by VDM (ie, 20% increase in surface area). Agreement of binary growth assessments between clinical measurements and VDM was determined by using the Cohen κ statistic. Interrater agreement of aortic segmentations was assessed using the Dice similarity coefficient and average Hausdorff distance to assess the mean distance between segmentations at the aortic boundary. To assess interrater agreement of surface area ratio, the mesh values from each rater's VDM analysis (unique segmentations) were mapped to a common aortic geometry to allow for direct comparison. *P* < .05 was indicative of a significant difference for all statistical tests. Statistical analyses were performed using Stata 14.0 (StataCorp).

## Results

### Patient Characteristics and VDM Analysis Failures

Of the 50 patients undergoing imaging surveillance of TAA on CT scans, five were excluded for lack of electrocardiographically gated CT acquisition, one was excluded for lack of thin-section reconstructions, two were excluded for poor aortic opacification, two were excluded for interval surgical aortic repair, and three were excluded for severe motion artifact. A total of 38 unique patients encompassing 105 CT angiograms and 68 surveillance intervals were selected for analysis. Among the 38 patients included for analysis, 3D growth mapping with VDM was successful in 35 (92%), and VDM analysis was successful in 58 of 68 (85%) surveillance intervals. Reasons for registration failure identified included irregular section intervals in source Digital Imaging and Communications in Medicine images (*n* = 3), excessive motion or stair-step artifacts (*n* = 2), streak artifacts from dense superior vena cava contrast (*n* = 2), and streak artifacts related to superior vena cava cardiac implantable electronic device leads (*n* = 3). Examples of error cases are shown in Figure E1 (online).

The mean patient age was 69 years ± 9 (age range, 46–85 years), and most patients were female (*n* = 21, 55%). The majority of TAAs involved the ascending aorta (*n* = 26, 68%) and were considered degenerative in origin (*n* = 23, 60%). Approximately one-third of patients (11 of 38) had a history of prior aortic repair and were undergoing postsurgical surveillance. Complete patient characteristics are shown in the Table. A median of two CT angiograms were obtained per patient (IQR, two to three angiograms; range, two to seven angiograms) with a median surveillance interval of 1.1 years (IQR, 1.0–2.0 years; range, 0.4–11.8 years).

**Table:**
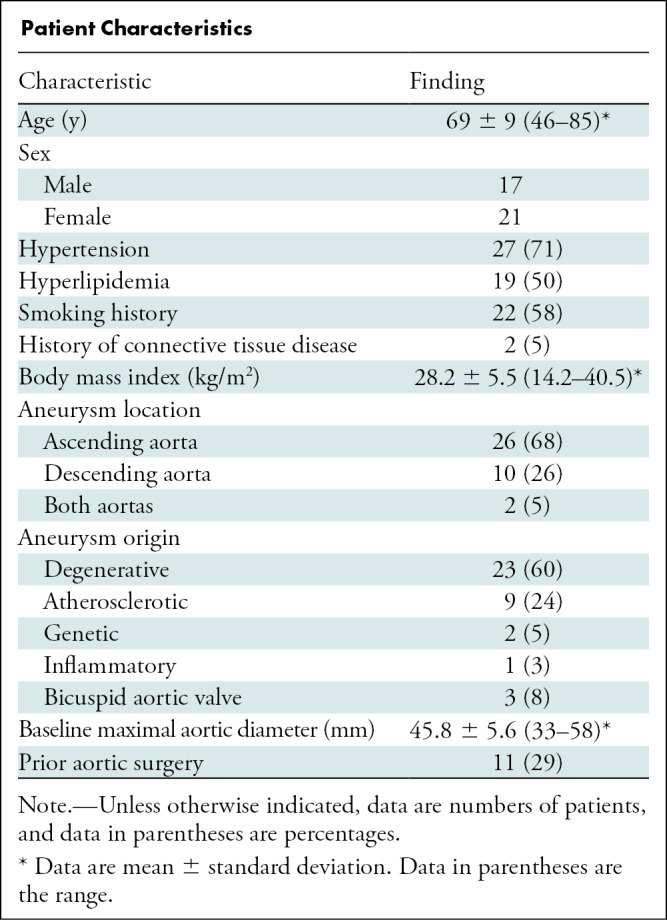
Patient Characteristics

### Registration Accuracy and Interrater Reproducibility Analysis

A total of 199 unique landmarks were manually placed at discrete anatomic locations along the aortic wall in 79 CT angiograms with a mean of 7.2 landmarks per patient. Considering all registration combinations, a total of 1021 point-pairs were used to assess landmark registration error. The median registration error was 0.77 mm (IQR, 0.54–1.10 mm; range, 0.07–4.57 mm; Figure E2 [online]).

**Figure 2:**
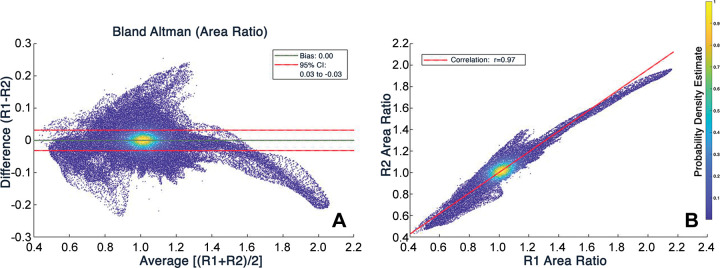
Interrater agreement analyses based on homologous surface mesh elements (approximately 5.4 million) generated from vascular deformation mapping. **(A)** Bland-Altman plot with bias and 95% CI depicting interrater agreement for area ratio. **(B)** Scatterplot shows strong correlation of area ratio values between raters. Color scale depicts probability density estimate at each point.

Interrater agreement for aortic segmentation was high, with a mean Dice similarity coefficient of 0.97 ± 0.02 (range, 0.93–0.99) and an average Hausdorff distance of 0.12 mm ± 0.20 (range, 0.01–1.20 mm). When comparing the interrater agreement of area ratio values between approximately 5.4 million homologous surface elements, we found no bias (bias = 0.0), narrow limits of agreement (-0.03 to 0.03 area ratio; Bland-Altman plot in Fig 2A), and excellent interrater correlation of area ratio values (*r* = 0.97; 95% CI: 0.97, 0.97; Fig 2B).

### Three-dimensional Growth Assessment with VDM

**Figure 3:**
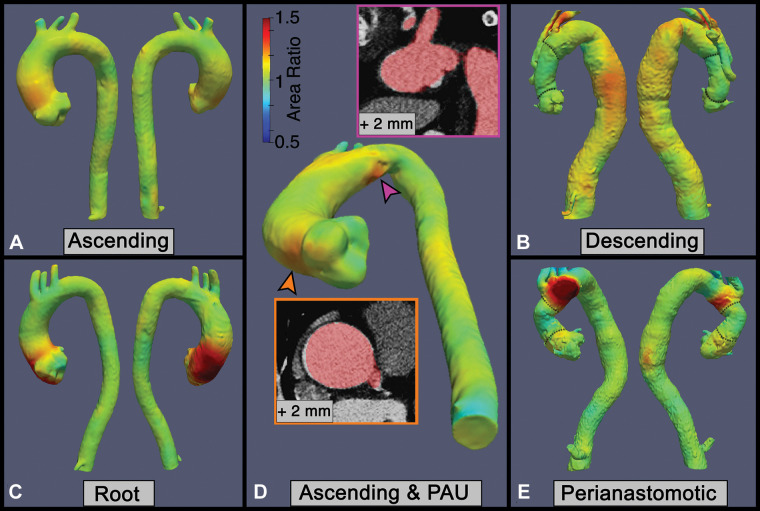
Representative examples of thoracic aortic aneurysm growth patterns identified with vascular deformation mapping in a clinical cohort of patients undergoing CT angiography imaging surveillance. **(A)** Circumferential growth involving the tubular segment of the ascending aorta. **(B)** Diffuse growth of the aneurysmal descending aorta in a patient with prior ascending aorta and aortic arch repair. **(C)** Eccentric growth of the aortic root and proximal ascending aorta. **(D)** Eccentric growth in the proximal tubular ascending aorta (orange arrowhead) and focal growth in the aortic arch at the location of a small penetrating atherosclerotic aneurysm (pink arrowhead). **(E)** Growth of the native aortic arch in a perianastomic distribution occurring 2 years after surgical replacement of the ascending aorta. Red masks depicting the baseline anatomy are overlaid on follow-up CT scans after rigid registration to allow for visual depiction of growth. Dotted lines in **B** and **E** indicate graft anastomoses. PAU = penetrating atherosclerotic ulcer.

**Figure 4:**
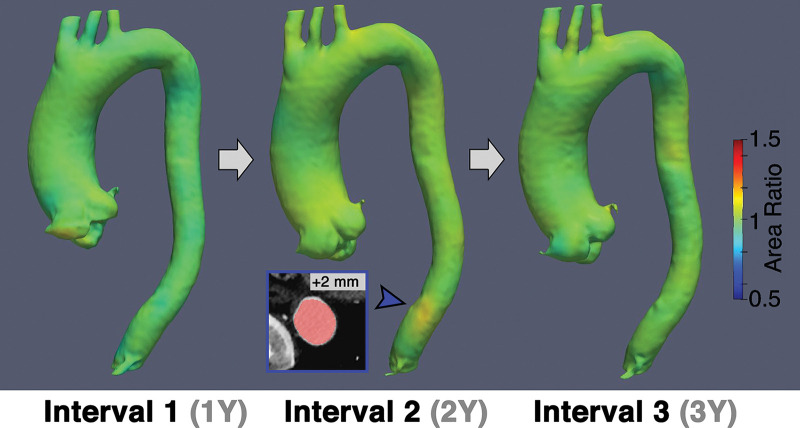
Representative images in a patient with a 4.7-cm aneurysm in the ascending aorta who demonstrated stability of the ascending aorta over three surveillance intervals totaling 6 years by vascular deformation mapping assessment. There was no growth of the ascending aorta according to three-dimensional assessment across all surveillance intervals; however, a small focal region of growth was detected at the distal descending level in interval 2 (arrowhead).

**Figure 5:**
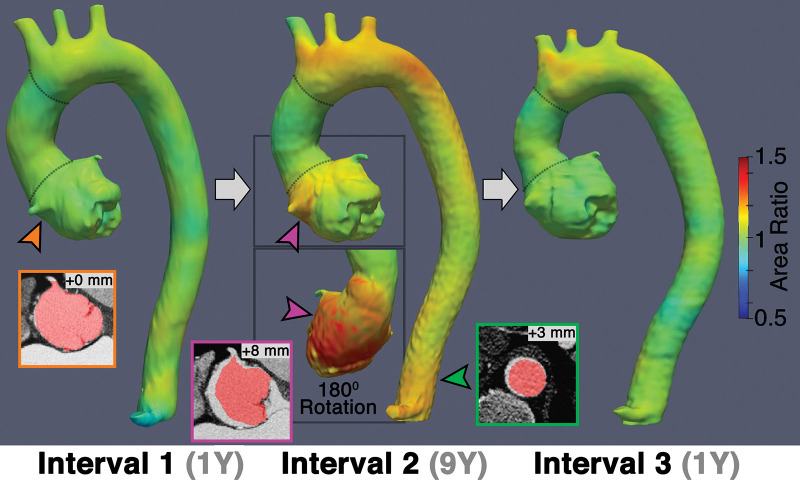
Representative vascular deformation mapping (VDM) assessment of a patient with Marfan syndrome who underwent valve-spring root and ascending repair, demonstrating stability of the root (orange arrowhead) at interval 1 (first interval after surgery). At interval 2, VDM showed progressive growth of the root (pink arrowhead), arch, and distal descending aorta (green arrowhead), with growth in the arch persisting at interval 3. Red masks depicting the baseline anatomy are overlaid on follow-up CT scans after rigid registration to allow for visual depiction of growth. Dotted lines indicate graft anastomoses.

Overall, the median area ratio assessed with VDM was 1.13 (IQR, 1.10–1.19; range, 1.05–1.78), and growth was detected with VDM in 14 of 58 (24%) intervals (defined as peak area ratio ≥1.2). VDM analysis clearly depicted aortic growth in common TAA locations including the ascending aorta (Fig 3A), descending aorta (Fig 3B), aortic root (Fig 3C), and perianastomotic distribution (Fig 3E). The location of growth by peak area ratio was localized to a segment of maximal aortic dilation in nine of 14 intervals (64%). In six of 14 (36%) intervals, VDM depicted growth outside the segment of maximal dilation (four in the aortic arch, two in the descending aorta). None of these six areas of submaximal growth were detected with clinical diameter measurements. Furthermore, changes in 3D aortic growth during imaging surveillance were clearly visualized with VDM (Figs 4, 5). Among the 14 patients who had more than one surveillance interval, 11 of 14 (78%) had stable aortic dimensions with VDM at all surveillance intervals (Fig 4), two of 14 (15%) had progressive growth at every interval, and one of 14 (7%) demonstrated stability at the initial surveillance interval and growth at subsequent intervals (Fig 5).

### Agreement between VDM and Clinical Diameter Measurements

**Figure 6:**
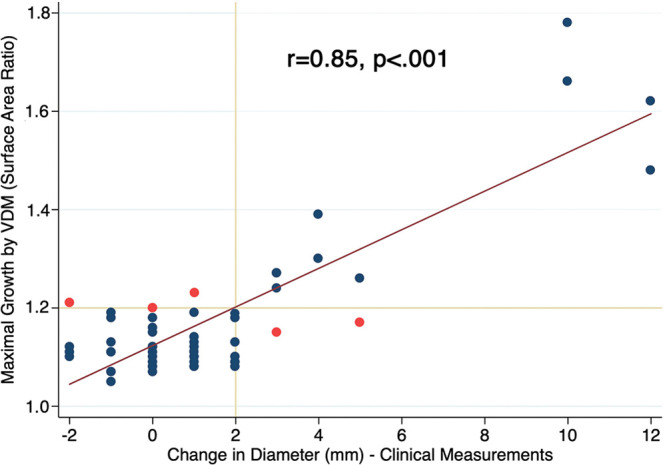
Scatterplot depicts agreement between maximal aortic growth quantification by clinical diameter measurements and vascular deformation mapping (VDM) (area ratio) at the aneurysmal segment. Red • depicts cases with discrepant growth assessments, whereas blue • represents concordant assessments.

There was strong agreement (*r* = 0.85; 95% CI: 0.75, 0.91; *P* < .001) between peak area ratio values and the change in maximal aortic diameter with clinical CT (Fig 6). When analyzing growth as a binary outcome, there was agreement between VDM and clinical diameter growth categorizations in 89% (49 of 55) of surveillance intervals (κ = 0.70; 95% CI: 0.42, 0.86). Clinical diameter change was not able to be determined in three surveillance intervals because baseline diameter was not clinically reported. Among the six intervals where growth assessments were discordant between VDM and clinical diameter measurements, there were four intervals where VDM indicated growth but diameter measurements did not and two intervals where diameter measurements indicated growth but VDM did not. In three of the four discrepant intervals with growth indicated by VDM, the location of peak area ratio was at the sinotubular junction, while the location of the clinically reported maximal diameter was at the midascending level. In six surveillance intervals, VDM analysis revealed an additional region of growth (≥1.2 area ratio) outside the maximally dilated segment, five of which were located in the arch (three arch-penetrating atherosclerotic ulcers, one proximal left subclavian artery, one fusiform dilation of the mid-arch), and one at the location of a small descending TAA PAU (Fig 4, interval 2).

## Discussion

In this article, we present results to support validation of a method for three-dimensional (3D) thoracic aortic aneurysm growth quantification using vascular deformation mapping (VDM) in a clinical cohort of patients with various manifestations of thoracic aortic aneurysm (eg, ascending, descending, and postsurgical) commonly encountered in clinical practice. In summary, we found that VDM analysis was technically successful in 85% of the evaluated intervals and that the most common reasons for failure of the VDM analysis included artifacts related to streak and motion artifacts at the ascending aorta. Despite small degrees of interrater variability in aortic segmentations, the final surface area ratio from VDM analysis showed excellent interrater agreement. In addition to quantifying 3D aortic growth in the maximally dilated segment, VDM identified additional regions of growth outside the primary aneurysmal segment in approximately one-third of patients. Lastly, while VDM demonstrated agreement with diameter growth assessments in the majority of cases (89%), the 3D nature of VDM allows for a more comprehensive depiction of the extent and distribution of growth along the aortic surface than is possible with diameter measurements.

Aortic diameter is the current metric used to assess growth and determine candidacy for surgical repair. However, diameter measurements vary and are limited in their ability to enable prediction of progressive growth and acute complications, such as aortic dissection (6,11). Assessment of aortic growth is a primary objective of imaging surveillance, enabling an indirect assessment of aortic wall integrity, information about the trajectory of disease progression, and likelihood of need for future surgical intervention (4,18). However, confident assessment of growth via aortic diameter measurements is often difficult, and measurement variability alone can occasionally result in growth assessments that erroneously suggest the need for surgical repair (5). The VDM technique represents an attempt to overcome such limitations by harnessing the high-spatial-resolution and volumetric (plane-independent) nature of CT angiography data in combination with deformable image registration techniques that are capable of registering CT images with submillimeter accuracy (17). The interrater variability of VDM surface area measurements in this study (±0.03) was 18% of mean values of surface area ratio change in our cohort (0.17). This degree of variability is substantially lower than described with clinical diameter measurements (±1–2-mm measurement variability relative to 1–2 mm of growth), suggesting that VDM may substantially improve the reliability and precision of aortic growth measurements despite the additional analysis time required in the current iteration of this algorithm. While aortic diameter has a clear relationship with tensile wall stress (ie, law of Laplace), this relationship assumes a circular shape, uniformly distributed and unidirectional stresses, and homogeneous composition of the aortic wall, assumptions that are not accurate in the TAA setting. Thus, kinematic assessment of aortic surface area changes with VDM may more accurately reflect underlying wall stresses due to the localized and multidirectional nature of the assessment.

Beyond providing a reproducible assessment of growth, the 3D nature of VDM allows for a more comprehensive evaluation than two-dimensional aortic diameter measurements. Quantitative mapping of TAA growth allows for investigation of unique parameters (eg, eccentricity, longitudinal extent, multifocality) that are otherwise unable to be easily captured. While VDM represents one of the first techniques for quantitative mapping of disease progression in TAA, similar image analysis techniques using deformable image registration have been used to phenotype and assess progression of diseases of the lungs (19,20), brain (21–23), and bones (24,25). The development of similar quantitative methods to assess TAA progression promises to improve risk stratification by more clearly separating intervals with slow versus no growth and may serve as a metric to better assess the effects of pharmacologic and surgical interventions. Preliminary investigations have suggested that VDM analysis may be able to aid surgical planning (13) and may help investigate the mechanisms of aortic dissection initiation (26,27).

Our study had several limitations. First, we did not systematically investigate the association of VDM metrics with patient outcomes, which will require larger cohorts with longitudinal follow-up. Second, while VDM analysis was technically successful in 92% of surveillance intervals, the technique is susceptible to errors in the presence of streak and motion artifacts. Thus, the performance of VDM may be suboptimal at centers that do not routinely use electrocardiographic gating and those that have older-generation CT scanners with narrower detector arrays, limiting generalizability. Third, VDM analysis currently requires more time than diameter measurement (20–30 minutes for manual segmentation and 20 minutes for registration); however, the overall analysis time can be mitigated by deep learning techniques for automated aortic segmentation (28). Lastly, given that we analyzed clinical CT angiography data, there is no available ground truth by which to adjudicate discrepant growth assessments between VDM and clinical diameter assessments.

In conclusion, vascular deformation mapping (VDM) is a reproducible method for comprehensive three-dimensional (3D) quantification of longitudinal aortic growth in a heterogeneous cohort of patients with thoracic aortic aneurysm. VDM analysis yielded reliable growth assessments in most surveillance intervals with excellent interrater reproducibility. Failure of this new method was predominantly related to streak and motion artifacts. Accurate quantitative 3D assessments of aortic growth may enable a more nuanced assessment of patient risk, disease phenotypes, and growth trajectories and may serve to better inform surveillance intervals, treatment decisions, and outcomes in patients with thoracic aortic aneurysm (TAA). However, given the low complication rate and slow growth of TAA, defining the prognostic and clinical importance of VDM measurement changes in aortic surface area requires further investigation in larger cohorts of patients with long-term follow-up.
